# Screening for tuberculosis and testing for human immunodeficiency virus in Zambian prisons

**DOI:** 10.2471/BLT.14.135285

**Published:** 2015-02-01

**Authors:** Katie R Maggard, Sisa Hatwiinda, Jennifer B Harris, Winifreda Phiri, Annika Krüüner, Kaunda Kaunda, Stephanie M Topp, Nathan Kapata, Helen Ayles, Chisela Chileshe, German Henostroza, Stewart E Reid

**Affiliations:** a Centre for Infectious Disease Research in Zambia, 5032 Great North Road, PO Box 34681, Lusaka, 10101, Zambia.; b University of Alabama at Birmingham, Birmingham, United States of America.; c National Tuberculosis and Leprosy Control Programme, Ministry of Health, Lusaka, Zambia.; d Zambia AIDS Related Tuberculosis Project, Lusaka, Zambia.; e Zambia Prisons Service, Ministry of Home Affairs, Kabwe, Zambia.

## Abstract

**Objective:**

To improve the Zambia Prisons Service’s implementation of tuberculosis screening and human immunodeficiency virus (HIV) testing.

**Methods:**

For both tuberculosis and HIV, we implemented mass screening of inmates and community-based screening of those residing in encampments adjacent to prisons. We also established routine systems – with inmates as peer educators – for the screening of newly entered or symptomatic inmates. We improved infection control measures, increased diagnostic capacity and promoted awareness of tuberculosis in Zambia’s prisons.

**Findings:**

In a period of 9 months, we screened 7638 individuals and diagnosed 409 new patients with tuberculosis. We tested 4879 individuals for HIV and diagnosed 564 cases of infection. An additional 625 individuals had previously been found to be HIV-positive. Including those already on tuberculosis treatment at the time of screening, the prevalence of tuberculosis recorded in the prisons and adjacent encampments – 6.4% (6428/100 000) – is 18 times the national prevalence estimate of 0.35%. Overall, 22.9% of the inmates and 13.8% of the encampment residents were HIV-positive.

**Conclusion:**

Both tuberculosis and HIV infection are common within Zambian prisons. We enhanced tuberculosis screening and improved the detection of tuberculosis and HIV in this setting. Our observations should be useful in the development of prison-based programmes for tuberculosis and HIV elsewhere.

## Résumé

**Objectif:**

Améliorer la mise en œuvre des dépistages de la tuberculose et du virus de l'immunodéficience humaine (VIH) de l'Administration pénitentiaire de la Zambie.

**Méthodes:**

Nous avons mis en œuvre les dépistages de masse à la fois pour la tuberculose et le VIH chez les détenus et le dépistage communautaire des personnes résidant dans les campements adjacents aux prisons. Nous avons également établi des systèmes de routine – en utilisant les détenus pour éduquer leurs pairs – pour le dépistage des nouveaux détenus ou des détenus symptomatiques. Nous avons amélioré les mesures de contrôle des infections, augmenté la capacité diagnostique et promu la sensibilisation à la tuberculose dans les prisons de Zambie.

**Résultats:**

Pendant une période de 9 mois, nous avons testé 7638 personnes et diagnostiqué 409 nouveaux patients atteints de tuberculose. Nous avons testé 4879 personnes pour le VIH et diagnostiqué 564 cas d'infection. En outre, 625 personnes avaient déjà été préalablement dépistées comme étant séropositives pour le VIH. En tenant compte des personnes déjà traitées contre la tuberculose au moment du dépistage, la prévalence de la tuberculose rapportée dans les prisons et les campements adjacents – 6,4% (6428/100 000) – est 18 fois supérieure à l'estimation de la prévalence nationale de 0,35%. Dans l'ensemble, 22,9% des détenus et 13,8% des individus résidant dans les campements étaient séropositifs pour le VIH.

**Conclusion:**

Les infections par la tuberculose et le VIH étaient courantes dans les prisons zambiennes. Nous avons amélioré le dépistage de la tuberculose et la détection de la tuberculose et du VIH dans ces conditions. Nos observations devraient être utiles pour développer les programmes pénitentiaires de lutte contre la tuberculose et le VIH ailleurs dans le monde.

## Resumen

**Objetivo:**

Mejorar la aplicación del servicio de prisiones de Zambia de detección de la tuberculosis y las pruebas del virus de la inmunodeficiencia humana (VIH).

**Métodos:**

Para la tuberculosis y el VIH, se llevó a cabo una detección masiva de reclusos y la detección comunitaria de aquellos que residían en campamentos adyacentes a las prisiones. También se establecieron sistemas rutinarios para la detección de los reclusos recién ingresados o sintomáticos en los que los reclusos actuaban como educadores de sus compañeros. Se mejoraron las medidas de control de la infección, se aumentó la capacidad diagnóstica y se promovió el conocimiento de la tuberculosis en las prisiones de Zambia.

**Resultados:**

En un periodo de 9 meses, se examinó a 7638 personas y se diagnosticó tuberculosis a 409 pacientes nuevos. Se realizaron pruebas del VIH a 4879 personas y se diagnosticaron 564 casos de infección. Previamente se había hallado que otras 625 personas era VIH positivas. Teniendo en cuenta aquellas personas que ya estaban en tratamiento para la tuberculosis en el momento de la detección, la prevalencia de la tuberculosis en las prisiones y los campamentos adyacentes, a saber, 6,4 % (6428/100 000), es 18 veces la estimación de la prevalencia nacional de 0,35 %. En total, el 22,9 % de los reclusos y el 13,8 % de los residentes del campamento eran VIH-positivos.

**Conclusión:**

Tanto la tuberculosis como la infección por VIH son comunes en las prisiones de Zambia. Se consiguió mejorar la detección de la tuberculosis y del VIH en este contexto, y esperamos que nuestras observaciones sean útiles para el desarrollo de programas de prisión de la tuberculosis y el VIH en otros contextos.

## ملخص

**الغرض:**

تحسين تنفيذ تحري السل واختبار فيروس العوز المناعي البشري من قبل مصلحة السجون في زامبيا.

**الطريقة:**

قمنا، فيما يتعلق بكل من السل وفيروس العوز المناعي البشري، بتنفيذ تحري واسع النطاق للسجناء، وتحري على نطاق المجتمع المحلي للأشخاص الذين يقيمون في معسكرات متاخمة للسجون. وقمنا كذلك بإنشاء نظم روتينية - اضطلع فيها النزلاء بتثقيف زملائهم - من أجل تحري النزلاء الجدد أو من توجد لديهم أعراض. وقمنا بتحسين تدابير مكافحة العدوى وزيادة القدرة التشخيصية وتعزيز الوعي بالسل في السجون في زامبيا.

**النتائج:**

قمنا خلال فترة 9 أشهر بتحري 7638 شخصاً وتشخيص 409 مريضاً جديداً بالسل. وأجرينا اختبار فيروس العوز المناعي البشري على 4879 شخصاً وقمنا بتشخيص 564 حالة إصابة. وتبين إيجابية 625 شخصاً آخرين لفيروس العوز المناعي البشري في السابق. وكان معدل انتشار السل الذي تم تسجيله في السجون والمعسكرات المتاخمة - 6.4 % (6428/100000) - 18 ضعف تقدير مدى الانتشار على الصعيد الوطني الذي بلغ 0.35 %. وبشكل عام، كان 22.9 % من النزلاء و13.8 % من الأشخاص الذين يقيمون في المعسكرات إيجابيين لفيروس العوز المناعي البشري.

**الاستنتاج:**

يشيع انتشار كل من السل وعدوى فيروس العوز المناعي البشري في السجون في زامبيا. وقمنا بتعزيز تحري السل وتحسين كشف السل وفيروس العوز المناعي البشري في هذه البيئة. وينبغي الاستفادة من ملاحظاتنا في وضع برامج للسجون بخصوص السل وفيروس العوز المناعي البشري في مناطق أخرى.

## 摘要

**目的:**

改进赞比亚监狱服务部门对结核病筛查和艾滋病病毒（HIV）检测的实施。

**方法:**

在结核病和艾滋病方面，我们都执行了大规模犯人筛查，并对监狱附近营地居民实施以社区为基础的筛查。我们还建立了常规系统，让狱友作为同伴教育者，筛查新入狱或有症状的囚犯。我们改进感染控制措施，增强诊断能力，提升赞比亚监狱结核病意识。

**结果:**

在9个月的时间中，我们筛选了7638名个人并诊断409例新结核病患者。我们对4879位个人进行艾滋病毒检测并诊断出564例感染。另外还有625人曾经检查出艾滋病毒阳性。包括那些在筛查时已经在接受结核病治疗的人，在监狱和邻近营地记录的肺结核患病率达6.4%（6428/10万人），这是全国发病率估算值0.35%的18倍。总体来看，22.9%的囚犯和13.8%的营地居民是艾滋病毒阳性。

**结论:**

结核病和艾滋病毒感染在赞比亚监狱都是常见的。我们加强结核病筛查并改善在此环境中的结核病和艾滋病毒的检测。我们的观察对于在其他地方制定基于监狱的结核病和艾滋病计划应该会有帮助。

## Резюме

**Цель:**

Повысить эффективность проведения скрининга на туберкулез и тестирования на вирус иммунодефицита человека (ВИЧ) в пенитенциарной службе Замбии.

**Методы:**

Как для туберкулеза, так и ВИЧ был проведен массовый скрининг заключенных и жителей, проживающих в лагерных поселениях, прилегающих к тюрьмам. Также были организованы постоянно действующие мероприятия — где в роли просветителей выступали сами заключенные — для скрининга вновь поступивших заключенных или тех, у кого имелись соответствующие симптомы. В результате удалось добиться улучшения системы инфекционного контроля, повышения диагностического потенциала и роста осведомленности о туберкулезе в тюрьмах Замбии.

**Результаты:**

За период 9 месяцев был проведен скрининг 7638 лиц и диагностировано 409 новых случаев туберкулеза. Также было протестировано 4879 лиц на наличие ВИЧ и диагностировано 564 случая этого заболевания. Еще 625 лиц показали положительный результат анализа на ВИЧ ранее. Включая тех лиц, кто уже проходил лечение от туберкулеза во время проведения обследования, распространенность туберкулеза, зарегистрированная в тюрьмах и смежных с ними лагерных поселениях, составила 6,4% (6428/100 000), что в 18 раз превышало общенациональную статистику по распространенности данного заболевания на уровне 0,35%. В целом, 22,9% заключенных и 13,8% жителей соседних лагерных поселений являлись ВИЧ-инфицированными.

**Вывод:**

Как туберкулез, так и ВИЧ-инфекция являются распространенными заболеваниями в тюрьмах Замбии. Были усилены меры по обследованию заключенных на туберкулез и улучшено обнаружение туберкулеза и ВИЧ-инфекции в этих условиях. Эти наблюдения должны быть полезны при разработке программ по выявлению туберкулеза и ВИЧ-инфекции в тюрьмах и других местах.

## Introduction

The United Nations has stated that prisoners are entitled to receive health care of a similar standard to that received by non-prisoners.^1^ However, substandard health care and a high burden of illness remain common features of prisons in low- and middle-income countries.^2^^–^^4^ In Zambia’s prison population, prevalence of human immunodeficiency virus (HIV) and tuberculosis are believed to be far higher than national estimates – of 12.7% and 0.35%, respectively.^5^^–^^7^ According to the most recent estimates – done more than 10 years ago – 27% of Zambian prisoners are HIV-positive^8^ and 4% are bacteriologically positive for tuberculosis.^9^

In 2010, the Zambia Prisons Service, Zambia’s national tuberculosis and leprosy control programme and the Centre for Infectious Disease Research in Zambia jointly implemented the Enhancing TB Services in Zambian Prisons project, which was designed to maximize the detection of tuberculosis and HIV-infected people in six of Zambia’s 86 prisons. These prisons were chosen – by project staff in consultation with the Zambia Prisons Service – based on their population size, historical tuberculosis burden and proximity to the capital city of Lusaka (Table 1). Together, the six prisons held 30% of Zambia’s prisoners. Funding limitations prevented inclusion of all of the prisons in Zambia. This paper describes the implementation and results of the project. Details of the screening algorithms used and prevalence of HIV and tuberculosis recorded at one of the prisons covered by the project have already been published.^10^

**Table 1:** Targeted populations for tuberculosis screening and human immunodeficiency virus testing, by prison site, Zambia, 2010–2011

Prison site	Description	Target population, no.
Lusaka Central Prison	Medium security	1300
Livingstone Central Prison	Medium security	800
Kabwe Prison Complex	The complex consists of four prisons: one maximum security, one medium security, one for female prisoners and one low-security farm	2600
Encampments adjacent to the Lusaka Central Prison and Kabwe Prison Complex	Occupied by prison personnel and their families	3000

## Methods

The TB REACH initiative of the Stop TB Partnership financed the project via a grant of 1 million United States dollars.^11^ Although the project began in October 2010 and lasted for 12 months – covering 3 months of training and sensitization and 9 months of screening – it was preceded by 6 months of regulatory approval, planning, and procurement and 12 months of facility renovations. The implementation team consisted of 27 project staff: two clinical officers, ten HIV counsellors, one project coordinator, one assistant project coordinator, six nurses, one community outreach coordinator, two laboratory technicians, two microscopists and two radiographers.

The project had four main objectives: (i) to improve infection control and diagnostic capacity, (ii) to strengthen awareness of tuberculosis in the prison environment, (iii) to develop better systems for the screening of new inmates and the referral of those who became symptomatic while incarcerated, and (iv) to diagnose all tuberculosis and HIV infections, by conducting a single mass screening in the prisons and community-based screening in the encampments – and then refer patients for treatment. Activities were implemented in several phases across the different prison facilities and encampments. The total target population comprised 4700 inmates and 3000 encampment residents.

### Infection control and diagnostic capacity

Before the project was launched, no prison in Zambia had specific measures for infection control or any onsite capacity for diagnosing tuberculosis. Typically, prisoners with tuberculosis were held in a sick-cell with other ill inmates – including those with mental illness or HIV infection. We constructed a 20-bed tuberculosis isolation facility in Lusaka Central Prison, our most overcrowded facility. At three prisons, we renovated existing structures to create permanent onsite microscopy laboratories equipped with Primo Star iLED fluorescence microscopes (Carl Zeiss Microimaging, Oberkochen, Germany). To facilitate mass and community-based screening, we purchased a 6.1-m long semi-mobile container that had been custom-fitted for fluorescence microscopy and digital chest X-ray (Oldelft Benelux, Veenendaal, Netherlands).

### Tuberculosis awareness

To improve awareness and prevention of tuberculosis among inmates, prison personnel and encampment residents, we conducted educational outreach and training activities (Table 2). These included performances by drama groups, didactic presentations and door-to-door canvassing in the encampments. To demonstrate their support, prison officers-in-charge participated in these events.

**Table 2:** Educational outreach and training for tuberculosis screening and human immunodeficiency virus testing in Zambian prisons, 2010–2011

Targeted population	Activity		No. of individuals reached	Description
	Educational outreach	Implementation training		
All prison personnel, other encampment residents and inmates	Yes	No	14 736^a^	Outreach events held within prisons and adjacent encampments to introduce project and inform about ongoing screening activities
Selected prison personnel and selected other encampment residents	No	Yes	136	Training and retraining, 1–3 days, for those prison personnel and members of neighbourhood health committees who will assist with screening
Selected inmates	No	Yes	197	Training and retraining, 2–5 days, for the inmate peer educators who will assist with tuberculosis screening and for inmates in the drama groups who will assist with outreach
Prison clinicians and nurses	No	Yes	43	Training, 3–5 days, in the provision of HIV care and treatment, interpretation of chest X-rays and TB case documentation

HIV: human immunodeficiency virus; TB: tuberculosis.

^a^ Including inmates (*n* = 8911), prison officers, their families and other prison staff.

As the Zambia Prisons Service has a critical shortage of health personnel, project staff trained three groups of individuals to recognize individuals who have tuberculosis symptoms and to screen or refer such individuals for care. The three groups trained were: (i) lay prison personnel and members of neighbourhood health committees; (ii) inmate peer educators; and (iii) prison officers – one per prison – who were selected as site coordinators.

In each prison, inmate peer educators were selected by the officer-in-charge, through an internal process that did not involve the project implementation team. They were often previously trained as peer educators for HIV and granted greater responsibility within the prison hierarchy because of good behaviour.

### Routine entry screening and referral

All convicted inmates and remandees sent to Zambian prisons should be given a general physical assessment upon prison entry. However, such assessment is frequently missed because of human-resource and other constraints. We used inmate peer educators and prison personnel to implement a routine tuberculosis screening protocol for all incoming prisoners regardless of symptoms and a referral protocol for inmates who developed any cough, fever, night sweats and/or weight loss while incarcerated (Table 3). The peer educators were trained to identify new or symptomatic inmates, complete screening data collection forms and observe the collection of two morning sputum specimens from each inmate. The specimens were examined with fluorescence microscopy by a trained laboratory technician either at a newly renovated microscopy laboratory or at the closest Ministry of Health clinic. Any inmate found smear-positive for tuberculosis or smear-negative and symptomatic was taken to the nearest Ministry of Health clinic for the initiation of anti-tuberculosis treatment and, on some occasions, further investigation. Provision of HIV testing and counselling varied by site, depending on the availability of test kits and trained counsellors.

**Table 3:** Summary of procedures for tuberculosis screening and human immunodeficiency virus testing in, Zambian prisons, 2010–2011

Intervention	Targeted population	Prison site			Procedures^a^
		Lusaka Central	Kabwe Complex	Livingstone Central	
Routine entry screening and referral screening^b^	All incoming prisoners and those who developed symptoms while incarcerated	Yes	Yes	Yes	Collection of two early-morning spot samples of sputum for FM, recording of tuberculosis history, exposure and symptoms, opt-out HIV testing, and physical examination by clinical officer
Mass screening and comprehensive entry screening^c^	All inmates	Yes	Yes	No	Collection of two early-morning spot samples of sputum for FM, recording of tuberculosis history, exposure and symptoms, opt-out HIV testing, digital chest X-ray, physical examination by clinical officer, culture of one sputum sample, and drug susceptibility testing of positive cultures
Community-based screening	All prison personnel and other encampment residents	Yes	Yes	No	Recording of tuberculosis history, exposure and symptoms, opt-out HIV testing, digital chest X-ray, physical examination by clinical officer plus – if symptomatic or with abnormal X-ray – collection of two early-morning spot samples of sputum for FM, culture of one sputum sample, and drug susceptibility testing of positive cultures

FM: fluorescence microscopy; HIV: human immunodeficiency virus.

^a^ For all of the targeted populations, tuberculosis treatment was provided by the Zambian Ministry of Health. HIV treatment was provided by the Zambian Ministry of Health or by a local nongovernmental organization supporting prison health services.

^b^ HIV testing and physical examinations were not possible in all of the sites.

^c^ The procedures for the mass screening and comprehensive entry screening procedures were identical while these two types of screening were being implemented concurrently. Once the mass screening ended, the algorithm for routine entry and referral screening – rather than that for the comprehensive entry screening – was followed.

Tuberculosis treatment at the Ministry of Health clinics was provided by the national tuberculosis and leprosy control programme, using existing registration and treatment procedures. Inmates found smear-positive were isolated in a designated cell or, at Lusaka Central Prison, in the new tuberculosis isolation facility and assessed weekly by smear microscopy. They returned to the general prison population when smear-negative.

### Mass and community-based screening

To determine the extent of tuberculosis and HIV disease among the inmates – and to ensure all infected individuals were diagnosed and received treatment – we conducted mass tuberculosis screening and HIV testing at five prisons. One prison was omitted from this phase because of limited time and funding. Because of poor infection-control procedures within the prisons and the close contact between inmates and prison personnel, we also conducted community-based screening in the encampments where prison personnel and their families were living (Table 3).

During mass screening, the implementation team was assisted by trained lay prison personnel, inmate peer educators and members of the local neighbourhood health committees. Tents were used as temporary screening stations. Once every inmate in a prison had been screened, the entire operation – i.e. the tents, semi-mobile container and project staff – moved into the adjacent encampment for the community-based screening.

Within the prisons, inmate peer educators sensitized and organized inmates in groups of up to 50 individuals, observed collection of two morning sputum samples, led groups through pretest counselling for HIV, guided smaller groups through the screening stations and completed data collection forms. Members of neighbourhood health committees proceeded door-to-door in the encampments to educate residents about the available screening services and initiate screening procedures. Although the inmates’ participation in tuberculosis screening was compulsory, that of prison personnel and other encampment residents was voluntary.

For each individual screened, it took 2 days to test and provide results. All participants proceeded through separate screening stations to provide a symptom history, have a digital chest X-ray taken and undergo HIV counselling and testing. In the final screening station, a clinical officer performed physical examinations and reviewed symptoms, X-rays, HIV status and smear microscopy results. Every inmate provided two sputum samples, regardless of their symptoms. However, encampment residents were only asked to provide two sputum samples if they presented with at least one tuberculosis-related sign or symptom – including cough, fever, night sweats, or weight loss – or showed an X-ray abnormality consistent with tuberculosis – or both.

All sputum samples were examined by fluorescence microscopy and one sputum sample per individual was inoculated in liquid media (BD BACTEC MGIT 960 mycobacteria testing system, Sparks, United States of America) and on solid BBL Lowenstein–Jensen medium (BD Diagnostics). The species and drug susceptibility of the *Mycobacterium* in each positive sample were investigated in a GenoType MDR line-probe assay (Hain Lifescience, Nehren, Germany).

Individuals diagnosed with tuberculosis were referred to the nearest Ministry of Health clinic for initiation of treatment. Individuals who were later found to be culture-positive for *Mycobacterium tuberculosis* but not previously diagnosed were located, if possible, and also referred for treatment. Individuals found to have drug-resistant tuberculosis who could not be tracked by the project team were reported to the nearest District Health Office.

Provider-initiated HIV counselling and testing were delivered to inmates on an opt-out basis. All inmates received HIV pretest counselling according to Zambian national and World Health Organization guidelines. Throughout the project, special attention was paid to the vulnerable nature of the inmate population in the context of opt-out testing. Decision to test, testing and post-test counselling were conducted in private, by trained external counsellors who were not affiliated with any prison. Individuals tested positive were referred for HIV care and treatment either at a Ministry of Health clinic or a local nongovernmental organization supporting prison health services.

### Data collection and analysis

Project-specific forms were used to collect data on demographics, tuberculosis and HIV histories, symptoms and results of the physical examinations, chest X-ray and laboratory tests. Project nurses and data-entry personnel reviewed these forms for completeness before data entry. To measure the project’s impact on screening for tuberculosis, data on the notification of tuberculosis cases in prisons and in encampments for the 3 years before the project’s implementation and for the 9 months of implementation were extracted from the treatment registers of Zambia’s national tuberculosis and leprosy control programme and compared. For our data analysis, a tuberculosis case was defined as anyone who had been found bacteriologically positive by fluorescence microscopy for acid-fast bacilli or by culture for *M. tuberculosis *or had been clinically diagnosed with tuberculosis*. *Individuals who were clinically diagnosed had negative or missing microscopy and culture results, but received an empiric diagnosis of tuberculosis based on presentation of tuberculosis-related signs and symptoms or an X-ray abnormality consistent with tuberculosis – or both. 

We calculated the prevalence of HIV infection, bacteriologically**-**positive tuberculosis, clinically**-**diagnosed tuberculosis and total tuberculosis. For tuberculosis, we compared the case detection during the project’s implementation with that of the previous 3 years. We also compared case detection among inmates screened comprehensively upon entry to the prison – i.e. on the basis of symptoms, fluorescence microscopy, cultures, X-rays and physical examinations – with inmates screened routinely – i.e. on the basis of symptoms and fluorescence microscopy only. Prevalences and case detection levels were compared using *χ^2^* tests. Data analyses were conducted in SAS version 9.3 (SAS Institute, Cary, USA).

### Ethical review

The project was reviewed by the Biomedical Research Ethics Committee of the University of Zambia (001–03–11) and the Institutional Review Board of the University of Alabama at Birmingham (F101014011). Both institutions waived the need for informed consent as tuberculosis and HIV screening were identified as standard care. An inmate representative was present at the Institutional Review Board of the University of Alabama’s meeting when the project’s protocol was reviewed.

## Results

Project outcomes are shown in Table 4. Between January and September 2011, we screened a total of 7638 individuals for tuberculosis. Eighty-two of these individuals were on anti-tuberculosis treatment when they were screened. We diagnosed an additional 409 new patients with tuberculosis: 160 who were found bacteriologically positive – by fluorescence microscopy or culture or both – and 249 who were positive for tuberculosis on clinical grounds alone. We therefore observed 491 patients with active tuberculosis giving an overall prevalence of 6428 cases per 100 000 people (Table 5).

**Table 4:** Project objectives and outcomes for tuberculosis screening and human immunodeficiency virus testing in Zambian prisons, 2010–2011

Main objectives	Outcomes
Improve tuberculosis infection control and diagnostic capacity	Constructed one tuberculosis isolation facility in Lusaka Central Prison, renovated and equipped microscopy laboratories at Lusaka Central Prison, Livingstone Central Prison and the Kabwe Prison Complex, and procured a containerized digital X-ray and microscopy laboratory
Strengthen tuberculosis awareness among inmates and prison personnel and other encampment residents	14 736 individuals participated in educational outreach and 197 inmate peer educators, 98 prison personnel and 38 members of neighbourhood health committees were educated about tuberculosis and tuberculosis screening procedures
Establish routine entry and referral screening for tuberculosis	Screening established in all six prisons and 2401 inmates were screened routinely
Conduct mass and community-based tuberculosis screening and HIV testing	Mass screening covered the 3929 inmates in five of the prisons while community-based screening covered 1308 of the residents in two encampments

HIV: human immunodeficiency virus.

**Table 5:** Tuberculosis screening and human immunodeficiency testing results in Zambian prisons, 2010–2011

Result	Type of screening^a^				
	Mass	Community-based	Comprehensive entry	Routine entry	Referral
**Tuberculosis**					
No. screened	3929	1308	799	1432	170
On ATT at time of screening					
No.	69	4	6	0	3
% (95% CI)	1.8 (1.4–2.2)	0.3 (0.1–0.8)	0.8 (0.3–1.6)	0.0 (0.0–0.3)	1.8 (0.4–5.1)
Bacteriologically positive					
No.	111	10	29	5	5
% (95% CI)	2.8 (2.3–3.4)	0.8 (0.4–1.4)	3.6 (2.4–5.2)	0.3 (0.1–0.8)	2.9 (1.0–6.7)
Clinically diagnosed					
No.	178	20	14	6	31
% (95% CI)	4.5 (3.9–5.2)	1.5 (0.9–2.4)	1.8 (1.0–2.9)	0.4 (0.2–0.9)	18.2 (12.7–24.9)
All cases					
No.	358	34	49	11	39
% (95% CI)	9.1 (8.2–10.1)	2.6 (1.8–3.6)	6.1 (4.6–8.0)	0.8 (0.4–1.4)	22.9 (16.9–30.0)
**HIV**					
No. positive (tested)	907 (3691)	112 (810)	99 (504)	45 (372)	26 (127)
% positive (95% CI)	24.6 (23.2–26.0)	13.8 (11.5–16.4)	19.6 (16.3–23.4)	12.1 (9.0–15.9)	20.5 (13.8–28.5)

ATT: anti-tuberculosis therapy; CI: confidence interval; HIV: human immunodeficiency virus.

^a^ The procedures for the mass screening and comprehensive entry screening procedures were identical while these two types of screening were being implemented concurrently. Once the mass screening ended, the algorithm for routine entry and referral screening – rather than that for the comprehensive entry screening – was followed.

When we stratified the results by sex, we found that 1.2% (12/1008) of the females screened and 2.2% (148/6630) of the males had bacteriologically**-**positive tuberculosis. The corresponding values for clinically-diagnosed tuberculosis were 1.5% (15/1008) and 3.5% (234/6630), respectively. The mean age of individuals with bacteriologically-positive tuberculosis was 46 years, with clinically-diagnosed tuberculosis it was 53 years, and with no tuberculosis, 43 years. Of the 160 individuals found bacteriologically positive for tuberculosis, one (0.6%) had multidrug-resistant tuberculosis and four (2.5%) had tuberculosis that was resistant to isoniazid only.

**Fig. 1:**
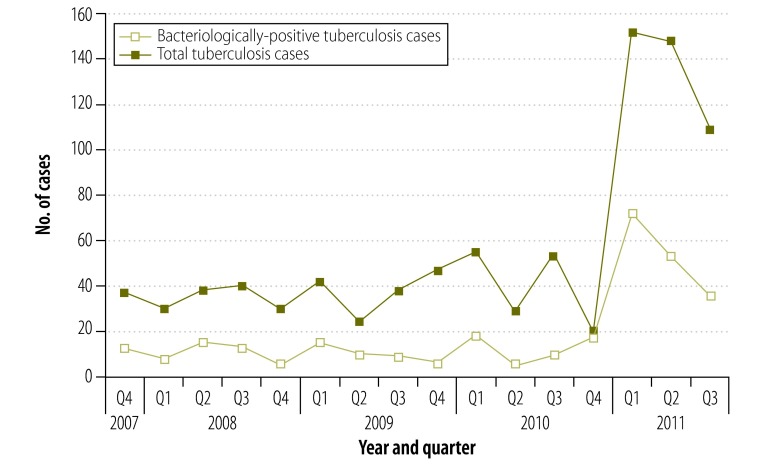
Numbers of tuberculosis cases recorded among inmates at six prisons and residents at two encampments, Zambia, 2007–2011

Note: Persons with sputum samples that tested positive for tuberculosis by microscopy or culture were defined as bacteriologically positive. Total tuberculosis cases include bacteriologically-positive and clinically-diagnosed tuberculosis patients. Individuals who were clinically diagnosed had negative or missing microscopy and culture results, but received an empiric diagnosis of tuberculosis because they presented with at least one tuberculosis-related sign or symptom or showed an X-ray abnormality consistent with tuberculosis – or both.

Between the end of 2007 and end of 2010 the numbers of diagnosed tuberculosis cases stayed fairly stable, however, these increased while the project was implemented (Fig. 1). For example, 138 cases of tuberculosis were recorded from January to September 2010 whereas 409 were recorded from January to September 2011.

Data source: Zambia’s national tuberculosis and leprosy control programme.

### Entry screening

Of 2231 inmates screened as they entered prison, 799 received comprehensive entry screening while the other 1432 received routine entry screening that only involved the assessment of symptoms and the examination of sputum smears by fluorescence microscopy. The recorded prevalence of bacteriologically-positive tuberculosis (3.6% versus 0.3%; *P* < 0.001) and clinically-diagnosed tuberculosis (1.8% versus 0.4%; *P* < 0.001) were both significantly higher with comprehensive screening than with routine screening (Table 5).

### HIV testing

Most (74%; 4694/6330) inmates and most (62%; 810/1308) encampment residents agreed to HIV testing or had a known prior status (Table 5). Testing resulted in 564 individuals (512 inmates and 52 encampment residents) being newly diagnosed with HIV infection. In addition, 625 individuals (565 inmates and 60 encampment residents) claimed and, in many cases, provided documentary evidence to show that they had previously been found to be HIV-positive. The overall prevalence of HIV infection was 22.9% (1077/4694) among the inmates, 13.8% (112/810) among the encampment residents, 37% (49/132) among the individuals with bacteriologically-positive tuberculosis, 37% (80/214) among the individuals with clinically-diagnosed tuberculosis, and 20% (1006/5081) among the individuals without tuberculosis.

### Tuberculosis treatment follow-up

Of the 409 individuals newly diagnosed with tuberculosis, 372 (91%) were recorded as initiating tuberculosis treatment. Of those who initiated treatment, 238 (64%) had a documented cure or treatment completion, 20 (5%) died, and seven (2%) defaulted. The remaining 107 (29%) had unknown outcomes because of their release from prison (*n* = 39), transfer to another prison (*n* = 47) or another reason (*n* = 21).

## Discussion

Mirroring the findings from prison studies in Cameroon, Côte d'Ivoire, Malawi, South Africa^2^^,^^12^^–^^15^ and several non-African countries,^16^^,^^17^ our results indicate that HIV infection and tuberculosis are more common within prisons than in the general population. In the targeted prisons and surrounding encampments, the prevalence of tuberculosis was 18 times higher than the Zambian national estimate.^6^ Similarly, HIV prevalence among the inmates we screened was nearly twice the Zambian national estimate.^5^

The Zambian prison health system has historically lacked the resources and capacity needed to implement rigorous screening, diagnosis and treatment measures for communicable diseases among inmates.^18^^–^^20^ The Enhancing TB Services in Zambian Prisons project demonstrates that – despite the multiple challenges of operating in the prisons of a lower middle-income country – a range of interventions can effectively enhance screening for tuberculosis and case detection for both tuberculosis and HIV. Although mass screening is resource-intensive and probably only possible on a periodic basis, we were able to test 3929 inmates and 1308 encampment residents in a 9-month period. In low-resource settings – where routine screening may otherwise be lacking – mass screening has value. The project also demonstrated the potential of more systems-oriented activities to address the chronic shortages of human resources for health in prisons – most notably the training and mobilizing of inmates as peer educators. The presence of trained inmate peer educators enabled the Zambia Prisons Service to overcome several barriers – e.g. lack of health personnel for routine symptom screening – and facilitated referrals and adherence support for those receiving treatment for HIV, tuberculosis or both infections.

During project implementation, several programmatic and clinical challenges were encountered. Despite the confined setting, loss of inmates to follow-up – both between tuberculosis diagnosis and treatment and during tuberculosis treatment – was a problem, primarily because the prisons had ineffective systems for tracking inmates once they had been released or transferred to other facilities. Despite strong support from the highest levels of the Zambia Prisons Service, the project also encountered resistance from some prison personnel, who saw the introduction of some activities – notably the routine entry screening and the supervision of inmate peer educators – as additional work for which they received no compensation. The recognition and mitigation of such resistance will be critical to the sustainability of most of the project’s activities.

As reported in other settings,^21^ the low sensitivity of the tools commonly used for tuberculosis screening and diagnosis was a challenge to the timely diagnosis of tuberculosis and, therefore, also a challenge to the timely initiation of treatment. In one of the prisons covered by the project, only 25% (22/88) of the patients with culture-positive tuberculosis were found sputum-smear-positive by fluorescence microscopy and 33% (29/88) of these patients never reported any symptoms.^10^ Such findings caution against the use of screening algorithms that triage inmates on the basis of their symptoms or smear results and they also highlight the need for more sensitive diagnostic tools. For prison and general populations alike, a rapid, accurate, point-of-care tool for the diagnosis of tuberculosis is urgently required – both to help curtail the disease’s spread and reduce its associated mortality.^22^

Since the project ended, the Zambia Prisons Service has continued routine entry and referral tuberculosis screening – with funding and ongoing technical assistance from the Centre for Infectious Disease Research in Zambia. Further assessment to understand the long-term cost of establishing routine screening – including the cost of training and supervising the inmate peer educators – is needed. However, the project showed that inmates can support better health-service delivery within prisons and that one-off mass screenings can be used to establish prevalence and initiate treatment. Descriptions of the project’s activities and findings also provide a basis for the adaptation of similar programmes in other prison services. Components that appeared critical to the success of the project’s activities are outlined in Box 1.

Box 1 **Designing prison programmes to screen for tuberculosis and test for human immunodeficiency virus**

- Engage high-level prison officials to facilitate buy-in of prison officers-in-charge and give legitimacy to the project.
- Secure start-up funding for monitoring, training, equipment, supplies and logistical support.
- Assess the capacity of – and strengthen – the existing care and treatment programmes, so that such programmes can cope with the additional cases detected.
- Consider carefully the ability of existing infrastructure to support infection control and onsite diagnostic capacity, while recognizing that renovations can be expensive and require a long time to complete.
- Train and empower inmates as peer educators who can conduct outreach and screening procedures, and develop peer-to-peer mentoring to maintain the cadre.
- Train prison personnel to supervise the inmate peer educators and facilitate their access to screening supplies and space.
- Prioritize the development of rapid and sensitive tools for tuberculosis diagnosis – within prisons and elsewhere.
- Conduct opt-out human immunodeficiency virus testing and counselling for all individuals screened for tuberculosis.
- Conduct entry and referral screening, with periodic screening at release, to assess the effectiveness of the interventions – instead of mass screening, which is resource-intensive.
- Encourage prison personnel and encampment residents to access tuberculosis screening, diagnosis, treatment and care.
- Develop a strategy to secure dedicated support and funding to promote sustainable, integrated health care for inmates.

The project – and our investigation of it – had several limitations. The activities and findings reported here are from only six prisons and may not be generalizable to all Zambian prisons. We may have underestimated tuberculosis prevalence because suboptimal sputum quality led to high frequencies of culture contamination.^23^ We had difficulty diagnosing extrapulmonary tuberculosis and we relied solely on fluorescence microscopy during routine entry screening. Conversely, the clinical misdiagnosis of tuberculosis in individuals who had other pulmonary disease may have led to tuberculosis prevalence being overestimated.

## Conclusion

In Zambia, many prisoners have tuberculosis, HIV infection or both, and various screening and treatment interventions could be implemented to reach a large number of prisoners in a comparatively short time. Our findings should act as a catalyst for improving prison services for the detection and treatment of tuberculosis and HIV infection in Zambia and elsewhere.
